# Twelve-years Old Girl with Retro-rectal Mass

**DOI:** 10.4103/1319-3767.49013

**Published:** 2009-04

**Authors:** Mounir Arroud, Chater Lamiae, Samir Atmani, Said Boujraf, My Abderrahmane Afifi, Moustapha Hida, Youssef Bouabdallah

**Affiliations:** Department of Pediatric Surgery, University Hospital Hassan II, Fez, Morocco; 1 Department of Pediatrics, University Hospital Hassan II, Fez, Morocco; 2 Biophysics Department, Faculty of Medicine and Pharmacy, Fez, Morocco

A 12-years-old girl followed for recurrent urinary infections was admitted to the pediatric department complaining of a 1-year history of constipation and pelvic pain. The child also reported of low spinal pain without any sensorymotor disorder. There were no events of rectal bleeding. Clinical examination revealed a suprapubic sensitivity with no perceptible abdominal mass. Digital rectal examination revealed a spherical, renitent and painless retrorectal mass. However, no intraluminal lesion was found. The anteroposterior and lateral X rays of both the thoracolumbar spine and the pelvis did not show any particularities. The abdominopelvic ultrasonography revealed a right laterouterine cystic mass measuring 4.5 cm in the largest diameter. The pelvic magnetic resonance imaging (MRI) showed an ovoid mass measuring 65 × 50 mm, with a regular wall thickness of 2 mm. The lesion had a hypersignal content in both the T1- and the T2-weighted images, pushing the rectum and the respective neighbouring structures [Figure 1, Figure 2]. The uterus and the ovaries as well as the lumbar spine were normal.

**Figure 1:**
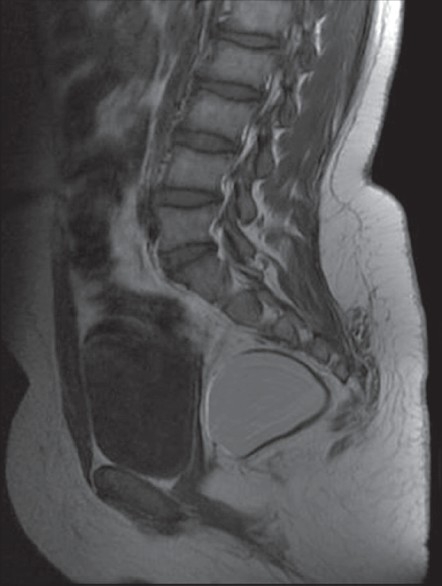
The pelvic T1-weighted magnetic resonance imaging in the sagittal slice showing a presacral mass with rectal compression

**Figure 2:**
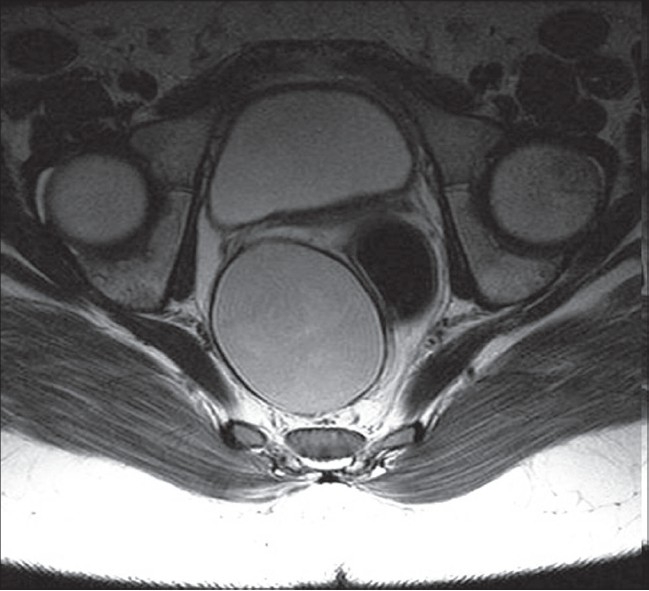
The pelvic T1-weighted magnetic resonance imaging in the transversal slice showing a presacral mass with cystic content

## QUESTIONS

1. What is your diagnosis?
2. What are your therapeutic options?

## ANSWERS

**Figure 3:**
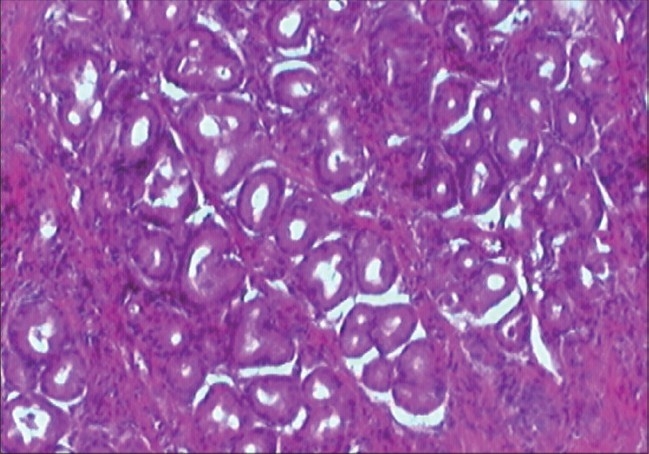
Highpower view of the heterotopic gastric mucosa

1. Rectal duplication with gastic mucosa heterotopy.
2. The surgical exploration through a sagittal posterior access found a retrorectal mass with a thick wall and greenish contents remaining in the meconium. Resection of the mass was performed. The histological examination revealed a digestive cystic duplication with a gastric body type of mucosa and submucosa [Figure 3]. The pos-operative course was uneventful and the patient made a good recovery.

## DISCUSSION

Duplication of the digestive tract is a rare malformation and the rectal localization represents 1–8% of this pathology.[1] The existence of a gastric heterotopy on this localization is exceptional, only eight cases of this association were reported in the literature.[2] Therefore, we aim at describing a new case of rectal duplication associated with heterotopic gastric mucosa and insist on the importance of the MRI for suggesting the diagnosis and the complete surgical excision of the mass that cures the recurrent symptoms and avoids the potential carcinomatous degeneration.

Rectal duplications usually present as a cystic mass in the retrorectal space but anterior localizations are also found.[3] Rectal duplications may communicate with the lumen of the rectum to form a diverticular rectal duplication or may be a separate cystic structure adjacent to the rectum as in this case.

The most frequently reported symptoms are pain and discomfort, constipation, urinary retention, recurrent perianal fistula, perianal infection and more rarely rectal prolaps. The gastic mucosa heterotopy can cause ulcerations and bleedings.[1,2,4] Cases of malignant degeneration of rectal duplication have been reported in adults.[5]

MRI allows precise diagnosis and its exact association. Scintigraphy, using TC99, is another useful diagnostic tool when gastric heterotopy is suggested.[2–4]

The appropriate treatment of rectal duplications is a complete surgical excision. It allows complete recovery and prevents potential malignant degeneration.[4,5]

Rectal duplication with gastric heterotopy is a full entity with variable presentation and complications. The diagnosis is suggested by MRI and confirmed by histological investigation. Total excision is the preferred form of treatment.
